# Repeat HIV testing of individuals with discrepant HIV self-test results in Central Uganda

**DOI:** 10.1186/s12981-019-0243-1

**Published:** 2019-09-12

**Authors:** Rose Kisa, Joseph K. B. Matovu, Esther Buregyeya, William Musoke, Caroline J. Vrana-Diaz, Jeffrey E. Korte, Rhoda K. Wanyenze

**Affiliations:** 1 Department of Disease Control and Environmental Health, School of Public Health, Makerere University, P.O. Box 7072, Kampala, Uganda; 2 Faculty of Health Sciences, Busitema University, Mbale, Uganda; 3 Research Department, Mildmay Uganda, Kampala, Uganda; 4 School of Public Health, Medical University of South Carolina, Charleston, SC USA

**Keywords:** HIV self-testing, Weak band, Confirmatory HIV testing, Uganda

## Abstract

**Background:**

According to the user instructions from the manufacturer of OraQuick HIV self-test (HIVST) kits, individuals whose kits show one red band should be considered to be HIV-negative, no matter how weak the band is. However, recent reports show potential for a second false weak band after storage, thereby creating confusion in the interpretation of results. In this study, we re-tested individuals whose results were initially non-reactive but changed to weak reactive results to determine their true HIV status.

**Methods:**

This study was nested within a large, cluster-randomized HIVST trial implemented among pregnant women attending antenatal care and their male partners in central Uganda between July 2016 and February 2017. Ninety-five initially HIV-negative respondents were enrolled into this study, including 52 whose kits developed a second weak band while in storage and 43 whose kits were interpreted as HIV-positive by interviewers at the next follow-up interview. Respondents were invited to return for repeat HIVST which was performed under the observation of a trained nurse counsellor. After HIVST, respondents underwent blood-based rapid HIV testing as per the national HIV testing algorithm (Determine (Abbot Laboratories), STAT-PAK (Chembio Diagnostic Systems Inc.) and Unigold (Trinity Biotech plc.) and dry blood spots were obtained for DNA/PCR testing. DNA/PCR was considered as the gold-standard HIV testing method.

**Results:**

After repeat HIVST, 90 (94.7%) tested HIV-negative; 2 (2.1%) tested HIV-positive; and 3 (3.2%) had missing HIV test results. When respondents were subjected to blood-based rapid HIV testing, 97.9% (93/95) tested HIV-negative while 2.1% (2/95) tested HIV-positive. Finally, when the respondents were subjected to DNA/PCR, 99% (94/95) tested HIV-negative while 1.1% (1/95) tested HIV-positive.

**Conclusions:**

Nearly all initially HIV-negative individuals whose HIVST kits developed a second weak band while in storage or were interpreted as HIV-positive by interviewers were found to be HIV-negative after confirmatory DNA/PCR HIV testing. These findings suggest a need for HIV-negative individuals whose HIVST results change to false positive while under storage or under other sub-optimal conditions to be provided with an option for repeat testing to determine their true HIV status.

## Introduction

HIV self-testing (HIVST), the process in which a person collects his or her own specimen, performs the test and interprets the results, is now recommended by the WHO [1] as one of the strategies that will improve access to HIV testing services. The specimens used for HIVST are either blood or oral mucosal transudate (OMT) which is used in the oral self-test (OraQuick ADVANCE Rapid HIV-1/2 Antibody test). HIVST provides a convenient alternative to tests initiated by a health worker [2], and it reduces the health providers’ workload in areas with a shortage of health resources [3]. Despite these benefits, questions have been raised on the performance of HIVST kits under sub-optimal field conditions such as those found in low and middle income countries [4, 5].

While some studies show a high level of stability of stored HIVST kits, others have observed that results change when results are read after storage. In 2016, Choko et al. [6] found that the majority of kits were still visible and easy to read after 28 days of storage under sub-optimal storage conditions. Only a few kits had their results changed, and this change was not related to storage conditions. However, a recent paper by Watson et al. [7] has shown that up to 29% of kits whose results were initially HIV-negative developed false weak-bands within 6 months of storage. Watson et al. [7] found that the earliest false weak bands developed after only 4 days of storage. These results suggest that individuals whose kits change from non-reactive to false weak-reactive results might not be able to tell what their true HIV status is. In the study by Watson et al., the kits were stored under varying storage conditions and the change in the results was not related to storage conditions.

Despite widespread use of rapid diagnostic tests (RDTs) in low and middle-income countries, they are prone to inaccuracy [8–10]. The inaccuracy is a result of one or more of the following factors: poor product performance, improper storage of test kits and supplies, clerical or transcription errors, and user errors in performing the test and/or interpreting the test result [11]. However, the post-marketing surveillance conducted to determine the performance of OraQuick on whole blood and oral fluid reported that the increased number of false-positive tests at one of the study site in Louisiana was not associated with any specific device characteristic, operator procedure or temperature condition [12]. Similarly, pre-incubating of HIV RDTs at 37 °C for 28 days did not substantially affect their diagnostic accuracy; and the initial results on OraQuick test kits remained stable over the 1-year follow up period in Malawi [6]. While oral OraQuick HIVST kits with less or equal to 1 month remaining to expiration had significant decline in specificity in California [13], three false negatives due to observer error were reported in UK [14], 0.25% in Ethiopia [15], 3.2% in Singapore [16], and 1.5% in India [17].

In line with manufacturer’s instructions, many researchers evaluating the performance of OraQuick HIVST kits have interpreted any two weak bands as HIV-positive [6, 18, 19]. However, no studies have been conducted to-date to explore if the development of a second false weak band on previously non-reactive kits implies a different HIV status for the person who initially tested HIV-negative. This study set out to investigate initially HIV-negative individuals whose kits developed a second weak band or whose kits were interpreted as HIV-positive by interviewers to determine their true HIV status.

## Materials and methods

### Study design and population

This cross-sectional study was nested within a larger cluster randomized controlled trial implemented among pregnant women attending antenatal care at three health facilities (Mpigi Health Center IV (HCIV), Entebbe Hospital and Nakaseke Hospital) in central Uganda. Details about the larger trial have been described elsewhere [20]. In brief, a total of 1514 pregnant women were randomly enrolled into the intervention (*n *= 777) or the control arm (*n *= 737). Intervention arm respondents received at least two HIVST kits (one for themselves, and the other for their male partner) while control arm respondents received information on the importance of male partner HIV testing but no HIVST kits were distributed. All respondents were followed for 3 months to assess differences in male partner HIV testing between the intervention and control arms. Intervention arm respondents were asked to return used HIVST kits to compare the results read by the client to the results read by a member of the study team. The main study was conducted between July 2016 and March 2017.

**Fig. 1:**
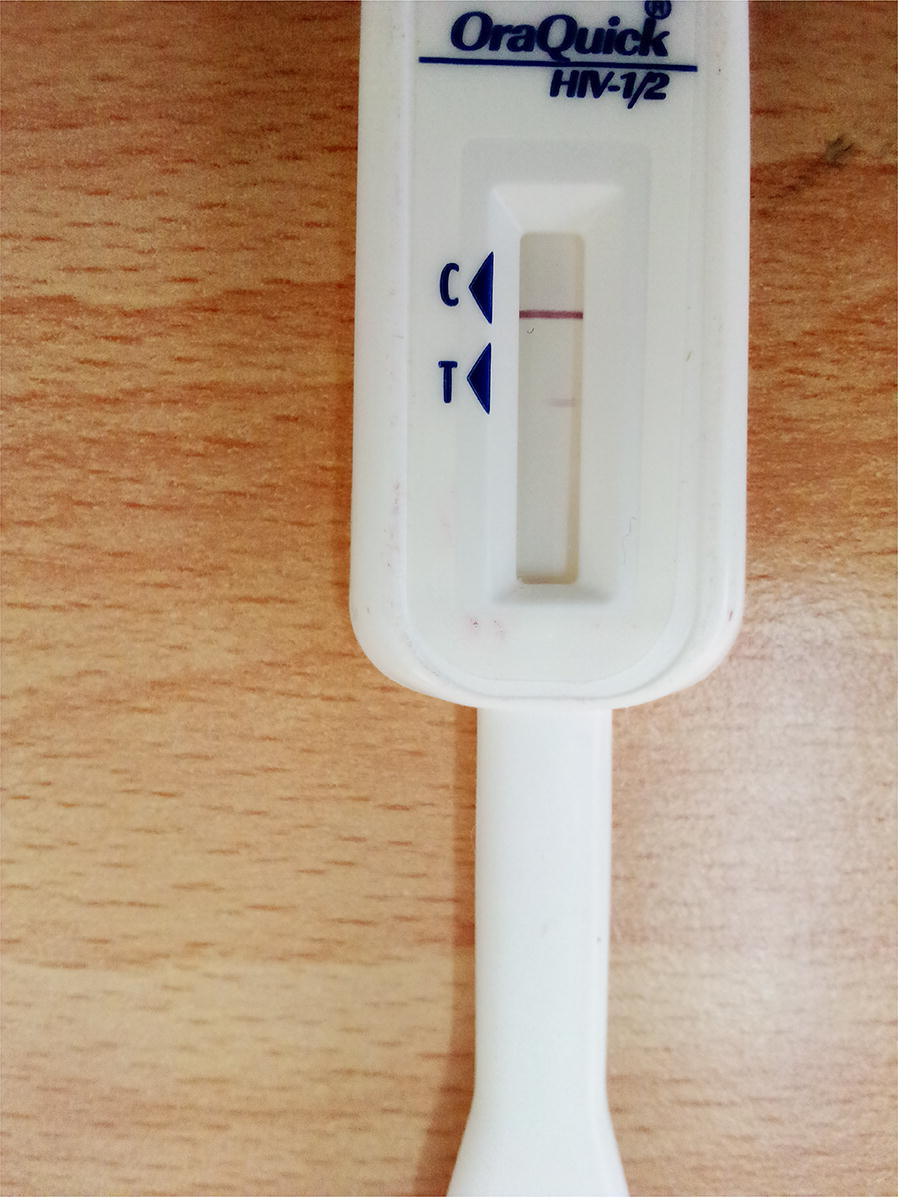
HIV self-kit that shows a faint second weak band in the ‘T’ area of the kit

This sub-study was conducted in March 2017 and enrolled 95 initially HIV-negative self-tested respondents including 52 (54.7%) whose HIV self-test kits developed a second weak band while in storage and 43 (45.3%) whose kits were interpreted as HIV-positive by the interviewers when the kits were returned to the health facility. It is important to note that all individuals who were enrolled in the larger study, including the 95 individuals enrolled in this study, were able to successfully read and interpret their HIVST results within the recommended time (i.e. between 20 and 40 min of performing the test). The individuals enrolled in this study read and interpreted their own HIVST results as HIV-negative. After performing HIVST, respondents were required to return used kits to the health facility to allow the study team to verify the results reported by the respondents but also to ensure that the kits were stored for future use. All returned used kits were re-read by the interviewers at the time of the follow-up interview (usually 1 month since the kits were used) and then stored. At the time of re-reading the kits, interviewers reported that they saw a second weak band on some of the kits (although clients reported that they saw one band, and interpreted their results as HIV-negative) and therefore interpreted the results as HIV-positive. This prompted the study team to check the other kits in the store and realized that some of the kits whose results had been reported as HIV-negative (by both the clients and interviewers) had developed a second weak band. The observation of a second weak band on the HIVST kits—either by the interviewers at the time of reading the results or while the kits were in storage—created doubts as to whether the respondents’ initial results were actually negative; warranting a need to contact them to return to undergo a series of tests to confirm their true HIV status. Figure 1 shows an example of a kit that originally had one band but which developed a faint second weak band.

### Study procedures

In line with the manufacturer’s instructions, HIVST results were interpreted as HIV-negative if only one “control” band was displayed or HIV-positive if two bands (“control” and “test”) were displayed, no matter how weak one or both bands were. Kits that showed no bands or whose testing area was discoloured were considered to be invalid. For this study, all initially HIV-negative individuals whose kits developed a second weak band while in storage, or whose kits were interpreted by the interviewer as HIV-positive at the next follow-up interview, were contacted by phone and invited to participate in a supervised repeat HIV testing exercise. Individuals were re-contacted between 1 and 4 months after they self-tested for HIV. HIV testing was done sequentially using three tests: HIVST under the supervision of a trained nurse counsellor; standard blood-based rapid HIV testing as per the national HIV testing algorithm and dry blood spots for DNA/PCR as the gold standard.

### Procedures for re-testing

After obtaining verbal consent, each respondent was given a pack containing one OraQuick^®^ Advanced Rapid HIV-1/2 Antibody Test (Orasure Technologies) self-test kit and manufacturer’s test instructions. Using an observation checklist developed by the study team, a trained nurse counselor observed and rated the self-tester’s performance on each of the HIVST steps as a quality control measure. The steps were in line with OraQuick instructions, namely: opening the bottle containing the buffer solution; putting the bottle with the buffer solution into the bottle-stand; swabbing the upper and lower gum; putting the test stick in the buffer solution; waiting for 20 min to read HIV test results; and interpreting HIV test results.

Sixty-two percent of respondents were able to perform all the six steps unsupported while the remaining 38% were supported by the nurse counselor to complete the repeat HIVST process. This phase was followed by clinic-based, rapid HIV testing which entailed drawing a blood sample for rapid HIV testing and a dry blood spot for DNA-PCR testing. Rapid testing used a serial algorithm of rapid HIV testing assays, which was the standard of care approved by the Ugandan Ministry of Health at the time. The algorithm included; Determine (Abbot Laboratories), STAT-PAK (Chembio Diagnostic Systems Inc.) and Unigold (Trinity Biotech plc.) as the tie breaker [21]. The process of obtaining samples was done in the facility laboratory located outside the antenatal clinic on the same day the respondents performed the repeat HIVST. The DNA-PCR test was used as a gold standard. Clearly labelled dried blood spots were delivered to the national reference laboratory using hub riders after 1 or 2 days of sample collection for upcountry sites, and daily for Entebbe hospital. The laboratory technologists at the facility were blinded to HIVST results; and at the national reference laboratory, both the oral fluid and rapid tests results were concealed.

### Data analysis

HIV testing data on the different tests were collected and entered in Microsoft Excel and analysis was conducted in STATA version 12. The proportions of the various test results on each of the different tests (repeat HIVST, blood-based rapid HIV tests and DNA/PCR) are presented.

## Results

### Respondents’ socio-demographic characteristics

Table 1 shows the socio-demographic characteristics of the 95 respondents (43 whose kits were read as HIV-positive by the interviewers and 52 whose kits developed a second weak band while in storage) enrolled in this study. Overall, 44 (46.3%) were males while 51 (53.7%) were females. Most of the respondents were aged 15–24 years (44.2%, 42/95); had secondary or higher education (32.6%, 31/95), and were currently married or cohabiting (94.7%, 90/95). All respondents self-reported that they had ever tested for HIV, with 96.8% (n = 92) reporting that their last HIV test results were HIV-negative. Of the remaining three respondents, one (1.1%) self-reported a previous HIV-positive result while 2 (2.1%) respondents indicated that they did not receive their previous HIV test results. It is important to note that all respondents read their HIV self-test results as HIV-negative immediately after HIV self-testing.

**Table 1:** Socio-demographic characteristics of 95 respondents whose HIVST kits had a second weak band by how the weak band was identified

Characteristics	How the second weak band was identified		Total (N = 95, %)
	Identified by the interviewer at follow-up interview^a^ (N = 43, %)	Observed while the kits were in storage (N = 52, %)	
*Sex*			
Male	24 (55.8)	20 (38.5)	44 (46.3)
Female	19 (44.2)	32 (61.5)	51 (53.7)
*Age*-*group*			
15–24	14 (32.6)	28 (54.9)	42 (44.2)
25–34	18 (41.9)	18 (35.3)	36 (37.9)
35+	11 (25.6)	5 (9.8)	16 (16.8)
*Education*			
Nursery/no formal education	14 (32.6)	8 (15.4)	22 (23.2)
Primary	11 (25.6)	15 (28.9)	26 (27.4)
Post-primary/vocational	8 (18.6)	8 (15.4)	16 (16.8)
Secondary	9 (20.9)	18 (34.6)	27 (28.4)
Tertiary	1 (2.3)	3 (5.8)	4 (4.2)
*Marital status*			
Currently married	4 (9.3)	9 (17.3)	13 (13.7)
Cohabiting	37 (86.1)	40 (76.9)	77 (81.1)
Never married/divorced/separated	2 (4.7)	3 (4.77)	5 (5.3)
Ever been tested for HIV, yes	43 (100)	52 (100)	95 (100)
*Result of the last HIV test*			
HIV-positive	0 (0.0)	1 (1.9)	1 (1.1)
HIV-negative	42 (97.7)	50 (96.2)	92 (96.8)
Did not receive results	1 (2.33)	1 (1.9)	2 (2.1)

^a^Interviewers reported that they saw a second weak band on the kits which the respondents had not seen

### Repeat HIVST, rapid HIV testing and DNA/PCR test results

Table 2 shows the overall results of the different HIV testing approaches to which the 95 respondents were subjected during the process of investigating their true HIV status. As shown, when the respondents were asked to undergo a repeat HIV self-test, 94.7% (90) were HIV negative, 2.1% (2) were HIV positive while results were missing for 3 respondents (3.2%). When the respondents were tested using a blood-based, rapid HIV testing kit, 97.9% (93) tested HIV-negative while 2.1% (2) tested HIV-positive. Finally, when the samples were subjected to DNA/PCR testing, 99% (94) tested HIV-negative while 1.1% (1) tested HIV-positive.

**Table 2:** Overall respondents’ HIV test results on repeat Oral HIVST, rapid HIV testing and DNA/PCR

HIV testing Approach	Repeat HIV test results			Total
	HIV-negative *n* (%)	HIV-positive *n* (%)	Missing *n* (%)	
Repeat oral HIV self-testing	90 (94.7)	2 (2.1)	3 (3.2)	95
Blood-based, rapid HIV testing	93 (97.9)	2 (2.1)	–	95
DNA/PCR testing	94 (99.0)	1 (1.0)	–	95

Table 3 shows results for respondents whose kits had a second weak band, stratified by HIV testing approach and how the second weak band was identified. Among the 43 individuals whose kits were read as HIV-positive by interviewers at the next follow-up visit, 39 (90.7%) tested HIV-negative on the repeat HIVST while 43 (100%) tested HIV-negative on both the blood-based, rapid HIV test and DNA/PCR test. On the other hand, among the 52 individuals whose kits developed a second weak band while in storage, 51 (98.1%) tested HIV-negative on the repeat HIVST; 50 (96.2%) tested HIV-negative on the blood-based, rapid HIV test while 51 (98.1%) tested HIV-negative on DNA/PCR. It should be noted that the percentage of respondents who tested HIV-negative was similar for repeat HIVST and DNA/PCR (98.1%). A slightly lower proportion (96.2%, n = 50) tested HIV-negative on the blood-based, rapid HIV test. When the analysis was restricted to only the DNA/PCR test (which was taken as the gold standard), we observed that all 43 individuals whose HIVST kits were identified as HIV-positive (due to a second weak band observed by the interviewers at follow-up) were HIV-negative while 51 (98.1%) of individuals whose kits developed a second weak band while in storage were also HIV-negative. When the DNA/PCR results were combined, we observed that 94 (99%) respondents were HIV-negative while only one respondent (1%) was HIV-positive.

**Table 3:** Repeat HIV test results stratified by HIV testing approach and how the second weak band was identified at the time of enrolment

HIV testing approach	Repeat HIV test results			Total
	HIV-negative *n* (%)	HIV-positive *n* (%)	Missing *n* (%)	
*Repeat HIV self*-*testing*				
Second weak band identified by interviewer at the follow-up interview	39 (90.7)	1 (2.3)	3 (7.0)	43
Second weak band identified after the kits were kept in the store	51 (98.1)	1 (1.9)	0 (0)	52
*Blood*-*based, rapid HIV testing*				
Second weak band identified by interviewer at the follow-up interview	43 (100)	0 (0)	0 (0)	43
Second weak band identified after the kits were kept in the store	50 (96.2)	2 (3.9)	0 (0)	52
*DNA/PCR testing*				
Second weak band identified by interviewer at the follow-up interview	43 (100)	0 (0)	0 (0)	43
Second weak band identified after the kits were kept in the store	51 (98.1)	1 (1.9)	0 (0)	52

## Discussion

Our study, which assessed the true HIV test results of initially HIV-negative individuals whose HIV self-test kits later developed a second weak band or were interpreted by interviewers as HIV-positive at the next follow-up visit, showed that almost all respondents had HIV-negative results across the three HIV tests: HIVST; blood-based, rapid HIV testing and DNA/PCR. Based on the results from the gold standard DNA/PCR test, we can confirm that 99% of initially HIV-negative respondents who were re-tested following the observation of a second weak band on their initial HIVST kits or whose kits were interpreted as HIV-positive by interviewers were HIV-negative.

Overall, 43 respondents were re-tested because of a discrepancy in the interpretation of results between the client and the interviewer, when the used test kits were examined at a follow-up interview. While respondents interpreted their results as HIV-negative immediately after HIV self-testing (i.e. within the recommended 20–40 min of the test); the interviewers interpreted the kits as HIV-positive after observing a second weak band at a later date. It is important to note that, at the follow-p visit, interviewers were expected to record the results on the kit into a prompted question. This was intended to check for the consistency of results read by the client with those of the interviewer. It was at this time that the interviewers interpreted the results on the kits as HIV-positive while the clients had originally interpreted them as HIV-negative.

When these individuals were re-tested as part of this study, we found that 91% of them tested HIV-negative on the repeat oral HIVST while all of them tested HIV-negative on both the blood-based rapid HIV test and DNA/PCR test. The relatively lower proportion of individuals testing HIV-negative on the repeat HIVST is largely due to the three participants who had missing results. Nevertheless, our findings suggest that all respondents whose kits were read as HIV-positive by interviewers were actually HIV-negative. The discrepancy in the interpretation of results could have been caused by the development of a second weak band after the respondents had interpreted their results as HIV-negative since interviewers were only able to read the kits after they were returned to the health facility. This was about 1 month since the kits were given to the pregnant women to take to their male partners. While previous studies from Malawi found a high level of stability of results on the HIVST kits after 12 months of storage [6, 22], a recent study by Watson et al. [7] has found that 29% of kits with true non-reactive results stored under different conditions changed to false weak reactive tests after 6 months of storage. The development of the second weak band in the study by Watson et al. was evident from as early as the fourth day of storage which suggests a high possibility that the kits that were interpreted as HIV-positive by interviewers in our study could have developed a second weak band by the time they were read. However, this is just a possible explanation. Further inquiry into the stability of results on HIVST kits over longer periods of storage is warranted.

It should also be recalled that 52 respondents who initially read their HIVST results as HIV-negative had their kits develop a second weak band after 1–4 months in storage. It is important to note that the observation of a second weak band on the stored kits was prompted by the interviewers’ reports at the follow-up visit. When interviewers reported seeing a second weak band on the kits, the study team decided to check the kits in storage. This is how the 52 kits were identified. When these individuals were subjected to repeat HIV testing, 98.1% were found to be HIV-negative on both the repeat HIVST and DNA/PCR tests while 96.2% were HIV-negative on the blood-based, rapid HIV tests. Since DNA/PCR was considered as the gold-standard test, our results suggest that 98% of the respondents who were re-tested were indeed HIV-negative. Only one person was confirmed as HIV-positive, possibly due to sero-conversion during the inter-testing period or because they were already HIV-positive but on antiretroviral therapy. It should be recalled that one respondent self-reported a previous HIV-positive result, and it is likely that this one person who tested HIV-positive on DNA/PCR could have been that one. However, these are just possibilities since we don’t know if the person who tested HIV-positive was on HIV treatment or whether he/she was the same person who self-reported a previous HIV-positive result.

## Strengths and limitations

Our study is the second one (after the one by Watson et al. [7]) to report results of individuals whose kits developed a second weak band after an initial HIV-negative self-test result. The study findings have implications on how to handle similar cases in the future as HIV self-test kits become more and more available in public health facilities in most low- and middle-income countries. However, unlike the study by Watson et al. [7], the implementation of this study was not part of the original study design. The need for this study emerged from the discrepancy in some of the results read by the clients and interviewers and the subsequent checking of kits in storage to identify if any other kits could have developed a second weak band. Nevertheless, our findings point to likely scenarios in the field settings as HIV self-testing becomes more and more popular in most countries. Finally, our results point to the need to advise individuals whose kits have developed a second weak band to undergo repeat HIV testing to confirm their true results.

## Conclusion

In conclusion, our results from repeat testing of individuals whose kits had a second weak band show that nearly all of them were HIV negative. Our results suggest a need to conduct repeat HIV testing among individuals with weak HIVST test bands to confirm their actual HIV status. However, since these results were obtained from a small post hoc study, we recommend bigger studies with external validity.

## Data Availability

The data will not be shared because it is institutional data but it can be made available on request.

## Abbreviations

DNA: deoxyribonucleic acid
HCIV: Health Centre IV
HIVST: HIV self-testing
OMT: oral mucosal transudate
PCR: polymerase chain reaction
RDT: rapid diagnostic tests
WHO: World Health Organisation
